# Giant filiform polyposis with high-grade dysplasia: A case report and review of the literature

**DOI:** 10.1016/j.amsu.2022.104433

**Published:** 2022-09-02

**Authors:** So Kasuga, Hiroyuki Anzai, Naohiro Makise, Hirofumi Sonoda, Yuzo Nagai, Shinya Abe, Yuichiro Yokoyama, Tsuyoshi Ozawa, Shigenobu Emoto, Koji Murono, Kazuhito Sasaki, Kazushige Kawai, Hiroaki Nozawa, Tetsuo Ushiku, Soichiro Ishihara

**Affiliations:** a Department of Surgical Oncology, The University of Tokyo, Tokyo, Japan; b Department of Pathology, The University of Tokyo, Tokyo, Japan

**Keywords:** Dsplasia, Ulcerative colitis, Inflammatory bowel disease, Crohn's disease, Case report

## Abstract

**Introduction and importance:**

Filiform polyposis, a rare condition also referred to as inflammatory polyposis or pseudopolyposis, is commonly observed in cases of inflammatory bowel disease, such as ulcerative colitis or Crohn's disease. It is generally considered a benign tumour characterised by multiple finger-like projections that are mostly observed in the transverse and descending colon.

**Case presentation:**

A 69-year-old woman with a history of ulcerative colitis for 18 years who underwent temporary decompression ileostomy for large bowel obstruction at another hospital was referred to our institution for further investigation. Abdominal computed tomography revealed bowel wall thickening of the transverse colon, and colonoscopy revealed stenosis in the hepatic flexure obstructing the endoscope. Although several biopsies of the tumour showed no malignancy, laparoscopic subtotal colectomy with lymph node dissection was performed. Histopathological findings revealed localised filiform polyposis with dysplasia.

**Clinical discussion:**

Filiform polyposis has been considered a benign inflammatory polyp without any risk of dysplasia. We accumulated previous cases of giant filiform polyposis and reviewed their characteristics. The presented case of filiform polyposis with ulcerative colitis complicated with high-grade dysplasia highlights the importance of considering malignancy in patients with filiform polyposis.

**Conclusion:**

In cases of giant filiform polyposis, even when no malignancy is detected, surgical resection should be considered for the possibility of a malignant component of dysplasia.

## Highlights

• This article is a case report and review of literature for filiform polyposis.
• Although filiform polyposis is generally considered a benign tumour, present case had dysplasia.
• This finding highlights the importance of considering malignancy in patients with filiform polyposis.

## 1 Introduction

Filiform polyposis is a polypoid change characterised by the elevation of the normal mucosa into finger-like projections. It was first described in 1974 by Appleman et al. [1]. Although it may be observed in the normal colon, it is more often associated with inflammatory bowel disease (IBD), such as ulcerative colitis (UC) [2,3]. Approximately 100 cases of filiform polyposis with IBD have been reported in PubMed. As filiform polyposis is considered a benign tumour, surgery is indicated when the patient experiences symptoms, such as melena or abdominal pain.

Here, we present a case of obstructive giant filiform polyposis (GFP) with high-grade dysplasia (HGD). Recognizing the possibility of malignancy such as that in the present case can improve the accuracy of diagnosis and treatment, thus benefiting patients in reducing hospital stay and related medical costs. We hereby declare that this work has been prepared and edited in accordance with the SCARE 2020 guidelines [4].

## 2 Presentation of case

**Fig. 1:**
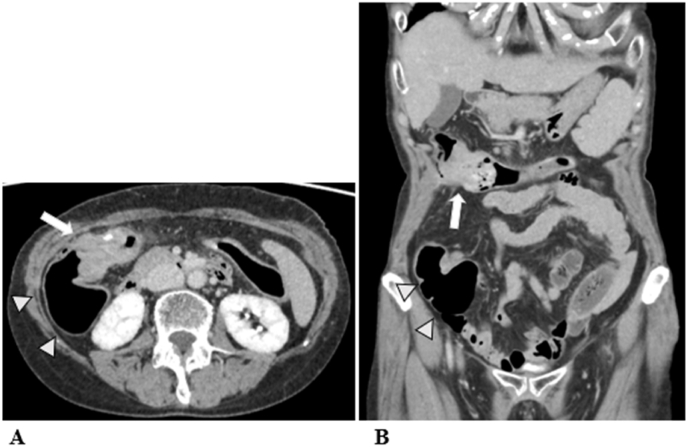
Computed tomography shows bowel wall thickening in the transverse colon (white arrows) and marked dilatation of the ileocecal part (white arrowheads). There are no enlarged lymph nodes or distant metastasis.

**Fig. 2:**
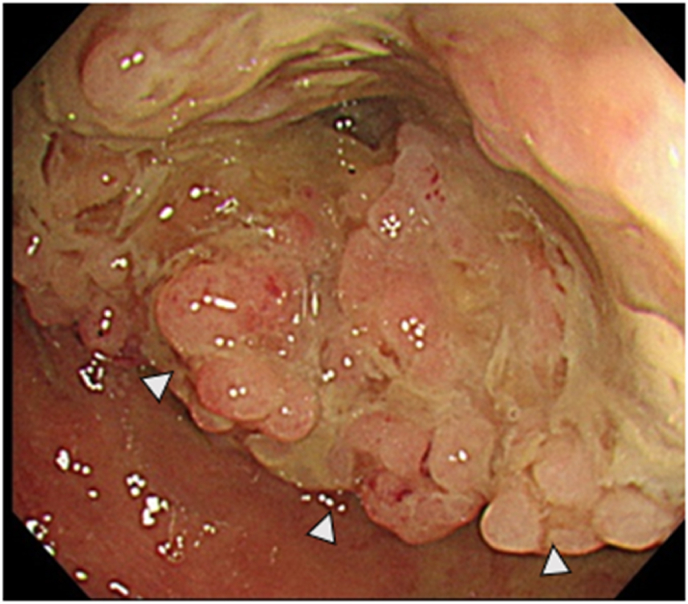
Macroscopic evaluation by colonoscopy shows multiple polypoid elevations in the transverse colon (white arrowheads).

**Fig. 3:**
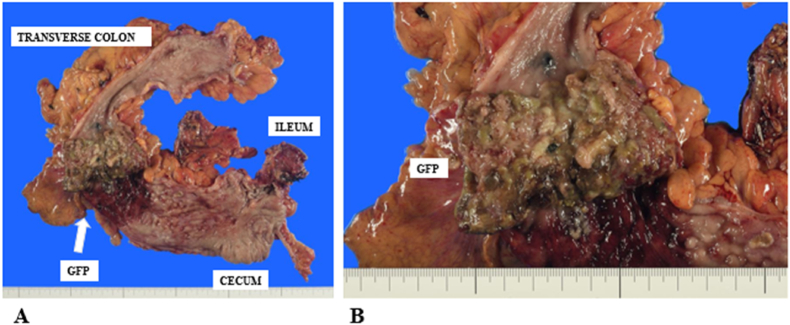
Macroscopic findings of the resected colon show a giant polyp in the transverse colon that measured 78 mm × 56 mm × 18 mm in size.

**Fig. 4:**
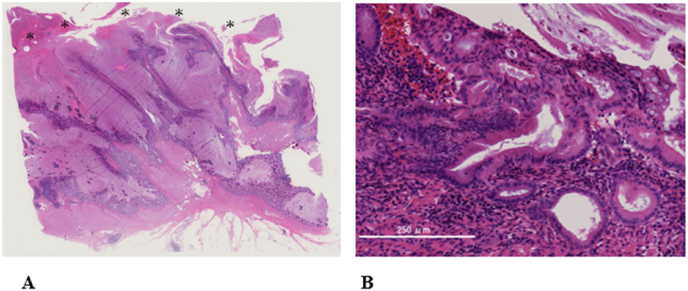
Microscopic views of the resected colon. A) Low-power view with the giant polyp shows that the polyp is composed of densely packed vermiform projections of the mucosa lined by non-neoplastic epithelium with thick mucin accumulation on the surface (asterisks). B) High-power view of the lesion with focal high-grade dysplasia.

A 69-year-old woman presented to hospital after an episode of rectal bleeding in December 1999. She underwent a colonoscopy, which revealed pancolitis. The patient was treated with prednisolone and 5-aminosalicylic acid and was clinically stable. After 2008, she was treated only with oral 5-aminosalicylic acid. In 2018, a surveillance colonoscopy revealed an inflammatory fibroid polyp at the transverse colon, which caused colonic stenosis. The polyp appeared similar to pseudopolyps, and the histopathology of the biopsy specimen revealed no malignancy. The patient was followed up closely to monitor the stenosis due to gradual narrowing of the transverse colon. In August 2020, she presented with intermittent right lower abdominal pain and was referred to hospital. Her medical history was not significant except for IBD. Her family history includes an older brother with a history of rectal cancer. Computed tomography (CT) performed 2 weeks later revealed marked distention of the ascending colon due to stenosis of the transverse colon. These findings suggested a large bowel obstruction, indicating the need for immediate decompression ileostomy. After surgery the patient recovered uneventfully. Although the diagnosis was not established, she was referred to our institution for further investigation. Laboratory investigations revealed normal serum haemoglobin levels. Tumour markers, such as carcinoembryonic antigen and carbohydrate antigen 19–9, were within the normal ranges, at 3.2 (normal range, 0–5.0) ng/mL and 21 (normal range, 0–37.0) U/mL, respectively. Serum anti-p53 antibody level was elevated to 2.85 (normal range, 0–0.40) U/mL. Abdominal CT revealed bowel wall thickening of the transverse colon (Fig. 1). No significant lymph node enlargement or distant or peritoneal metastases was observed. Fluorodeoxyglucose-positron emission tomography/CT showed an abnormal uptake with a maximum standardised uptake value of 7.4 in the transverse colon. Her previous colonoscopy findings from our institution revealed stenosis in the hepatic flexure, which obstructed the endoscope (Fig. 2). Several biopsies of the tumour showed no malignancy. Subsequently, the patient underwent laparoscopic subtotal colectomy with lymph node dissection with main feeding artery ligation at the tumour level. The surgically resected specimen showed a giant polyp measuring 78 mm × 56 mm × 18 mm, which was histologically examined (Fig. 3). Histopathological examinations revealed that the giant polyp was composed of densely packed vermiform projections of the mucosa lined by non-neoplastic epithelium with thick mucin accumulation on the surface (Fig. 4A). Based on this, the polyp was diagnosed as localised filiform polyposis. In addition, a small focus of HGD was observed at the base of the polyposis (Fig. 4B). All lymph nodes were negative for malignancy. The filiform polyposis mucosa showed no abnormal p53 expression by immunohistochemistry using anti-p53 antibodies. After confirming the histopathological results, ileostomy closure and ileorectal anastomosis were performed.

The postoperative course was uneventful, and she was discharged 10 days after surgery.

Six months after the last surgery, an ileo-rectal anastomosis was performed. Then, postoperative follow-up was scheduled a yearly outpatient setting and colonoscopy as well as after surgery of ulcerative colitis associated cancer.

## 3 Discussion

Recently, filiform polyposis was defined as an uncommon entity characterised by multiple finger-like polypoid colonic lesions on endoscopic and histopathological evaluation and submucosal fibrovascular accentuation and normal mucosa on histological examination. Although the mechanism of occurrence has not been clarified, the tumour is hypothesised to be formed by the isolation of faecal materials in “pockets” formed by the filiform polyps [2,[5], [6], [7], [8], [9]].

We conducted a MEDLINE search to identify previously reported cases of filiform polyposis. Approximately 100 cases of filiform polyposis have been reported in PubMed. Generally, it forms in the colon of patients with a history of IBD. Eighteen cases (full text available) of filiform polyposis originated from the normal colon with no history of IBD, of which two (11%) cases were associated with adenocarcinoma (Table 1) [2,[6], [7], [8],[10], [11], [12], [13], [14], [15], [16]]. Eighty-six cases of filiform polyposis were associated with IBD, of which, 48 and 38 were associated with UC and Crohn's disease (CD), respectively (Table 2) [3,[17], [18], [19], [20], [21], [22], [23], [24], [25], [26], [27], [28], [29], [30]]. As shown in Table 2, there were no noticeable differences in clinical features, such as location of occurrence, age of onset, and symptoms, between UC and CD. There was one case [17] of adenocarcinoma in UC (2.0%) and one case [17] of dysplasia in CD (2.6%). Clinicopathological findings of obstructive GFP cases with UC are summarised in Table 3 [3,[19], [20], [21], [22], [23], [24], [25], [26], [27]]. The most frequent site of occurrence was the transverse colon in eight of the 11 (72.7%) cases, as in this patient. The time interval from the initial diagnosis of UC to GFP formation ranged from 1 to 25 years, with varying degrees of inflammation. Therefore, the duration of UC or degree of inflammation was not associated with the development of GFP.

**Table 1:** Clinical features of reported cases of filiform polyposis without inflammatory bowel disease.

No	Reference	Year	Case	Age/sex	Symptoms	Location	Treatment	Pathology
1	Raila et al. [10]	1989	1	69/M	Haematochezia	T	Polypectomy	Benign
2	Cheng et al. [11]	1989	1	47/F	Abdominal pain	C	Polypectomy	Benign
3	Kang et al. [12]	2007	1	47/M	Asymptomatic	T	Polypectomy	Benign
4	Oakley et al. [2]	2007	1	50/M	Asymptomatic	Total colon	APR	Benign
5	Vainer et al. [13]	2007	1	37/F	Abdominal pain	A	Right-sided hemicolectomy	Benign
6	Lee et al. [6]	2010	7	37–81 /2F5M	Asymptomatic 3 Abdominal pain 2	All S	All polypectomy	All benign
7	Kim et al. [8]	2010	1	83/F	Abdominal pain	S	Segmental resection	Benign
8	Wolf et al. [14]	2011	1	45/M	Asymptomatic	T	Right-sided hemicolectomy	Benign
9	Mavrogenis et al. [15]	2013	1	31/M	Obstruction	A	Right-sided hemicolectomy	Benign
10	Boulagnon et al. [7]	2014	1	54/M	Anaemia	A	Right-sided hemicolectomy	Adenocarcinoma
11	Okuno et al. [16]	2019	1	59/M	Asymptomatic	S	Sigmoidectomy	Adenocarcinoma

M, male; F, female; T, transverse colon; C, cecum; A, ascending colon; S, sigmoid colon; R, right colon; APR, abdominoperineal resection.

**Table 2:** Literature review of giant filiform polyposis with ulcerative colitis or Crohn's disease.

	UC (n = 48)	CD (n = 38)
**Male** (%)	33 (68.8)	27 (71.0)
**Female** (%)	15 (31.3)	11 (28.9)
**Age** [median (range)]	36 (10–71)	40 (8–71)
**Disease duration** (median year)	3	5
**Presentation**		
Abdominal pain	15	15
Rectal bleeding, bloody diarrhoea	13	9
Colonic obstruction	11	3
Diarrhoea	4	4
Incidental finding	5	1
Abdominal mass	4	4
Anaemia	3	0
Weight loss	2	5
**Location**		
Transverse	22	13
Descending	9	10
Sigmoid	5	3
Rectum	3	0
Ascending	2	4
Cecum	2	1
Entire colon	3	3
ND	1	3
**Treatment**		
Total colectomy (procto-)	22 (1)	3
Subtotal colectomy	10	2
Colectomy	6	9
Segmental colectomy	5	17
Endoscopic resection	2	0
Medication	2	3
ND	0	4
**Pathology**		
Adenocarcinoma	1	0
Dysplasia	0	1

UC, ulcerative colitis; CD, Crohn's disease; ND, not detected.

**Table 3:** Cases of ulcerative colitis associated with obstructive giant filiform polyposis (11 cases).

No	Reference	Year	Age/Sex	Disease duration (year)	Disease status	Location	Size	Treatment	Pathology
1	Forde et al. [3]	1980	48/M	3	Active	T-D	ND	Subtotal colectomy →Proctocolectomy	Benign
2	Sonnino et al. [19]	1987	37/F	6	Active	T	23 cm	Total colectomy	Benign
3	Okayama et al. [20]	1996	54/M	2	Active	T, D	11.5 cm, 18 cm	Total colectomy	Benign
4	Hurlstone et al. [21]	2002	68/M	25	Inactive	R	ND	EPMR	Benign
5	Maldonado et al. [22]	2004	27/M	2	Inactive	T	5 cm	Subtotal colectomy	Benign
6	Yada et al. [23]	2005	32/F	1.2	Inactive	T	12 cm	Total colectomy	Benign
7	Maggs et al. [24]	2008	49/M	15	Inactive	T	ND	Subtotal colectomy	Benign
8	Ikeda et al. [25]	2011	71/M	3	Inactive	S	9 cm	Sigmoidectomy →APR	Benign
9	Nagashima et al. [26]	2013	25/M	2	Active	T, D	23 cm, 18 cm	Total colectomy	Benign
10	Rached et al. [27]	2018	20/M	1	Active	T	ND	Total colectomy	Benign
11	Our case	2020	69/F	22	Inactive	T	7.8 cm	Colostomy →Subtotal colectomy →Total colectomy	Dysplasia

M, male; F, female; T, transverse colon; D, descending colon; R, rectum; S, sigmoid colon; ND, not detectable; EPMR, endoscopic piecemeal mucosal resection; APR, abdominoperineal resection.

Surgery is often the treatment of choice in symptomatic cases, especially GFP cases, such as our case. In past reports of GFP cases, abdominal pain and gastrointestinal bleeding were the most common indications for surgery, with 30 cases of abdominal pain and 22 cases of gastrointestinal bleeding (Table 2). Because filiform polyposis is a benign tumour, the surgical strategy in UC is total colectomy, followed by subtotal colectomy, while that in CD is segmental colectomy. Although lymph node dissection was not discussed in previous studies, it is necessary to consider whether lymph nodes should be dissected when selecting a surgical technique because HGD was observed in this case; therefore, even if multiple preoperative biopsies show no dysplasia and the surrounding mucosa is normal without any signs of inflammation, malignant findings are not completely excluded. In this case, the large mass filled the entire lumen, obstructing passage through the bowel, making examination of the region proximal to the colon from the tumour difficult. The patient gradually developed abdominal pain, and obstruction was observed on CT. Given her compromised status, the patient was scheduled for a temporary loop ileostomy. The patient was discharged from the local hospital and was referred to our hospital for further investigation. The inflammatory mucosal tumour was resected, and a small amount of epithelial dysplasia was observed. We selected an appropriate surgical technique by initially performing the surgery for diagnostic purposes. In this case, immunohistochemistry using anti-p53 antibodies revealed that the dysplastic lesion did not show p53 abnormal expression. As p53 mutations appear to be the initiating mutation in inflammatory carcinogenesis, these findings do not support the inflammatory carcinogenesis of filiform polyposis [31,32]. Furthermore, dysplasia was observed in some of the polyps in this case with no background of inflammation, so evidence was insufficient to support inflammatory carcinogenesis. Interestingly, in our study, the frequency of cancer and dysplasia in non-IBD patients was rather high compared with that in patients with IBD-associated dysplasia. This further supports that the present case was not IBD-associated dysplasia. Furthermore, we performed a total colectomy; therefore, we could preserve the rectum with ileorectal anastomosis, whereas a total proctocolectomy would have been required for colitis-associated colorectal cancer.

Filiform polyposis has been considered a benign inflammatory polyp without any risk of dysplasia; however, a few cases of filiform polyposis associated with dysplasia and adenocarcinoma have been reported [7,[16], [17], [18]]. Although these reports suggest that filiform polyposis may have carcinogenic potential, the mechanism of carcinogenesis of filiform polyposis remains unclear; thus, further accumulation of cases is necessary. Limitations of this study include the inherent limitations of case reports, such as the inability to generalize, the inability to prove causality, and the risk of overinterpretation.

## 4 Conclusion

This case shows that it is crucial to consider the possibility of malignancy with filiform polyposis. Furthermore, even when no malignancy is detected in cases of GFP, we should consider the possibility of a malignant component of the dysplasia. The conventional resection of GFP requires careful consideration of the surgical technique and disease pathology. Nevertheless, further accumulation of data and investigation of filiform polyposis are warranted.

## Ethical approval

This study is a case report and is not considered to require an ethics application.

## Source of funding

The authors have no sources of funding for research.

## Author contribution

Study concept and design, acquisition and analysis of data, drafting of the work and surgery: So Kasuga, Hiroyuki Anzai. Drafting of the manuscript, critical revision of the manuscript for important intellectual relevance: Tetsuo Ushiku, Soichiro Ishihara. Revision of the manuscript: Munetoshi Hinata, Hirofumi Sonoda, Hirofumi Sonoda, Yuzo Nagai, Shinya Abe, Yuichiro Yokoyama, Tsuyoshi Ozawa, Shigenobu Emoto, Koji Murono, Kazuhito Sasaki, Kazushige Kawai, Hiroaki Nozawa. Study supervision: Soichiro Ishihara.

## Trail registry number

This study is a case report and is not considered a research study registration is not required.

## Guarantor

The guarantor is So Kasuga.

## Data availability statement

Data available on request. The data underlying this article will be shared on reasonable request to the corresponding author.

## Consent

Written informed consent was obtained from the patient for publication of this case report and accompanying images. A copy of the written consent is available for review by the editor-in-chief of this journal on request.

## Provenance and peer review

Not commissioned, externally peer reviewed.

## Declaration of competing interest

The authors have no conflicts of interest directly relevant to the content of this article.
